# A novel method of determining the active drag profile in swimming via data manipulation of multiple tension force collection methods

**DOI:** 10.1038/s41598-023-37595-y

**Published:** 2023-07-05

**Authors:** A. Haskins, C. McCabe, R. Kennedy, R. McWade, A. B. Lennon, D. Chandar

**Affiliations:** 1 School of Mechanical and Aerospace Engineering, Queen’s University Belfast, Belfast, BT9 5AH UK; 2 School of Sport, Ulster University, Belfast, BT15 1AP UK

**Keywords:** Engineering, Scientific data, Fluid dynamics, Data acquisition

## Abstract

A novel method aimed at evaluating the active drag profile during front-crawl swimming is proposed. Fourteen full trials were conducted with each trial using a stationary load cell set-up and a commercial resistance trainer to record the tension force in a rope, caused by an athlete swimming. Seven different stroke cycles in each experiment were identified for resampling time dependent data into position dependent data. Active drag was then calculated by subtracting resistance trainer force data away from the stationary load cell force data. Mean active drag values across the stroke cycle were calculated for comparison with existing methods, with mean active drag values calculated between 76 and 140 N depending on the trial. Comparing results with established active drag methods, such as the Velocity Perturbation Method (VPM), shows agreement in the magnitude of the mean active drag forces. Repeatability was investigated using one athlete, repeating the load cell set-up experiment, indicating results collected could range by 88 N depending on stroke cycle position. Variation in results is likely due to inconsistencies in swimmer technique and power output, although further investigation is required. The method outlined is proposed as a representation of the active drag profile over a full stroke cycle.

## Introduction

The velocity of a body swimming through water is dependent on the resultant of the horizontal forces acting on said body, with propulsive forces acting to accelerate a body and resistive forces acting to decelerate a body. Elite level swimming is a competitive sport, with the goal being to maximise the ratio of propulsive forces to resistive forces. For human aquatic locomotion, hydrodynamic resistance is one of the most important variables. Hydrodynamic resistance in swimming can be split into two components, namely active drag and passive drag. Active drag is the resistive force that acts on a swimmer when swimming through the water and is constantly changing during a stroke cycle^1, 2^. A stroke cycle in swimming is a movement that returns to its beginning and repeats itself in the same sequence^3^. Active drag is composed of frictional, pressure, and wave drag and is greatly influenced by the technique of each athlete; it can provide an insight into how an individual’s technique is influencing swimming velocity, potentially indicating how a particular change in technique can improve or negatively affect a swimmer’s performance. A thorough understanding of an individual’s active drag profile is necessary, especially at the elite level (where winning margins are frequently small), in order to optimise technique, minimise active drag and maximise forward velocity.

Passive drag is the resistance generated by a swimmer’s body whilst the swimmer moves through the water in a fixed position^1, 2^. Passive drag acts on the swimmer during the glide phases of the stroke, such as when streamlining off the wall or following a dive-entry^4^. Similar to active drag, passive drag is composed of frictional, pressure and wave drag. A definition of passive drag has been included as, although not being investigated in this study, passive drag forms an important variable in a number of existing active drag prediction methodologies currently in the literature.

### Active drag investigation methods

Active drag is difficult to measure directly, largely due to complex fluid motion and accessibility limitations, meaning researchers use indirect methods to estimate values of active drag. Typical values of active drag can range anywhere between 20 and 210 N, depending heavily on forward velocity^5^.

One well-known method for quantifying active drag is the Measurement of Active Drag (MAD) system, developed by Hollander^6^. The system functions with a swimmer applying a force against a series of underwater fixed pads, during the underwater pull phase of the stroke. A force transducer located at one end of the pool records the force applied by the swimmer on each pad. Assuming a constant swimming velocity, the propulsive force applied to the pads is considered as the average drag force acting on the swimmer^1^. An example of the MAD system is displayed in the work of Narita^7^.

There are accuracy limitations with the MAD system, as a swimmer’s technique must be adjusted to allow interaction with each pad, meaning an accurate reflection of drag associated with an individual’s typical technique is not provided^8^. The assumption of no acceleration during swimming is also erroneous, since velocity changes continuously during a typical stroke cycle due to the fluctuating interplay between propulsive and resistive forces^9^. The added resistance encountered by propelling off an abutment, a fixed pad, rather than water may artificially inflate pulling force results^10^. Due to the equipment layout, the MAD system is also only able to estimate active drag during front crawl swimming.

A further method to measure active drag is the Velocity Perturbation Method (VPM) which involves towing an object with known hydrodynamic drag behind a swimmer. The swimmer completes one swim with the object and one without; the difference in velocities between the two swims is used to find active drag, assuming the power output is constant between swims^11^. The reader is directed to the work of Webb and Banks^11^ for further mathematical explanation of the method. Equation (1) displays the calculation of active drag, ${F}_{r1}$, using a towed object of known resistance, ${F}_{b}$, and the measured velocity of the swimmer with, ${V}_{2},$ and without,${V}_{1},$ the towed object of known resistance.

1 $${F}_{r1}= \frac{{F}_{b}{V}_{2}{V}_{1}^{2}}{{V}_{1}^{3}-{V}_{2}^{3}}$$

Researchers have also used mathematical methods such as the Naval Architecture Based Approach (NABA)^11^. The NABA method is based on a self-propulsion experiment for ships investigating interaction between the propeller and naked hull. The reader is directed to the work of Webb and Banks^11^ for further explanation of the method. The naked hull resistance (${R}_{Naked}$) is found as a function of hull velocity with non-dimensional torque and thrust coefficients found as a function of advance ratio ($J$) of the propeller. The advance ratio, $J$, is displayed in Eq. (2)^11^, where $V$ is the advance velocity, $n$ is the number of revolutions per second and $D$ is the propeller diameter.

2 $$J=\frac{V}{nD}$$

Applied to human swimming, the arms act as a propeller and the remaining body as a hull. ${R}_{Naked}$ is found using a passive drag towing experiment, equal to the measured drag of the body at a known velocity. The athlete is then towed while swimming at artificially increased velocities towards the equipment.

$${(R-T)}_{Measured}$$, where $$R$$ is the hull resistance and $$T$$ is the thrust produced, is quantified during the experiment by measuring the resistance to tow when the athlete is swimming at these known artificially increased velocities. The advance ratio, $$J$$, must be kept constant at the various velocities of testing. The artificially increased towing velocities will see a higher drag due to increased velocity, as from the drag equation, visible in the referenced Morais’ Eq. (2)^12^. The naked hull resistance must be corrected for each towing velocity, using a correction factor ($${R}_{correction})$$, equal to the difference in drag at the higher tow velocity and free swimming velocity. Predicted resistance data is used to estimate the correction factor. The active drag is then calculated as follows^11^:

3 $${R}_{Active}= {(R-T)}_{Measured}- {\Delta R}_{Correction}+ {R}_{Naked}$$

Comparing active drag results between the VPM and NABA methods, there is agreement between both methods averaging around 130 N at velocities 5%, 10%, and 15% higher than free swimming velocity^11^. Although the NABA method and VPM produce similar results, the VPM has higher uncertainty and a higher standard deviation of results compared to NABA^11^. In addition, these methods only provide a single value of drag across the stroke cycle, meaning there is no active drag profile created over the full stroke cycle. An active drag profile would allow visualisation of how active drag changes over the full stroke, indicating the areas of maximum and minimum drag experienced by an individual.

Other active drag measurement methods include bioenergetics and modelling methods^1^. One bioenergetics method attempts to measure the changes in force production to overcome applied known force changes to the swimmer, by measuring the change in oxygen intake^13^. A graph of additional towing force against the change of functional parameter relative to unloaded level is plotted to find a regression line, with the functional parameter being oxygen consumption above rest level. An example of this approach is demonstrated by Di Prampero^13^ and Rumyanstev^1^, whereby the active resistance at a given swimming velocity, F_0_, is found at the horizontal axis intercept of the graph and this is equal to the active drag.

Experimental modelling methods determine the intermediate boundary postures of swimming phases and find the corresponding drag coefficients for each posture at different towing velocities^1, 14^. Values of swimming velocity at different boundary postures are predicted from synchronised video recordings of swimming and intracyclic velocity recordings. When the time and drag coefficients are known for each swimming phase, active resistance can be found. The value of calculated drag will differ from the real active drag as there is an acceleration in real swimming, unlike the constant velocity assumed in the modelling^1^. This difference is caused by the inertial force of added mass of water acting on the body. To overcome the inertia, the swimmer must expend a quantity of energy. Using Newton’s second law, the active drag may be subdivided into two forces:

4 $${F}_{DA}={F}_{DP}+\Delta ma$$

where ${F}_{DA}$ is active drag, ${F}_{DP}$ is passive drag and $\Delta ma$ is the added mass inertial force, with $m$ being mass of water and $a$ being the value of acceleration. The added mass value of water can be predicted as:

5 $${F}_{t}-{F}_{DP} =\left(m+\Delta m\right)a$$

where ${F}_{t}$ is the towing force^1^. This method has led to overestimated values of active drag at approximately double the value of passive drag, likely due to the full magnitude of passive drag being included in the calculation^1^.

In summary, the requirement of complex equipment that is frequently inaccessible and the lack of full stroke cycle active drag profiling has led to the development of a proposed novel method for calculating the active drag profile of a swimmer.

## Material and methods

### Participants

Six males, who have achieved qualification standards for Irish National level competition in the 200 m Freestyle (< 130.16 s or > 481 FINA Points) and one international level male triathlete, participated in the study (anthropometric details in Table 1).

**Table 1:** Anthropomorphic data of the athletes.

Age (yrs)	(20 ± 4)
Height (m)	(1.80 ± 0.18)
Chest depth (m)	(0.27 ± 0.03)
Shoulder width (m)	(0.5 ± 0.02)

### Ethics approval and consent to participate

This study was approved by the local Queen’s University Belfast ethics committee, namely the Engineering and Physical Sciences Faculty Research Ethics Committee, and was performed in accordance with the guidelines and regulations of the Declaration of Helsinki. All participants were provided with verbal and written explanations of the purpose, procedure and risks related to the study and provided written consent. Informed consent was obtained from all participants of the study.

### Procedure—equipment

**Figure 1:**
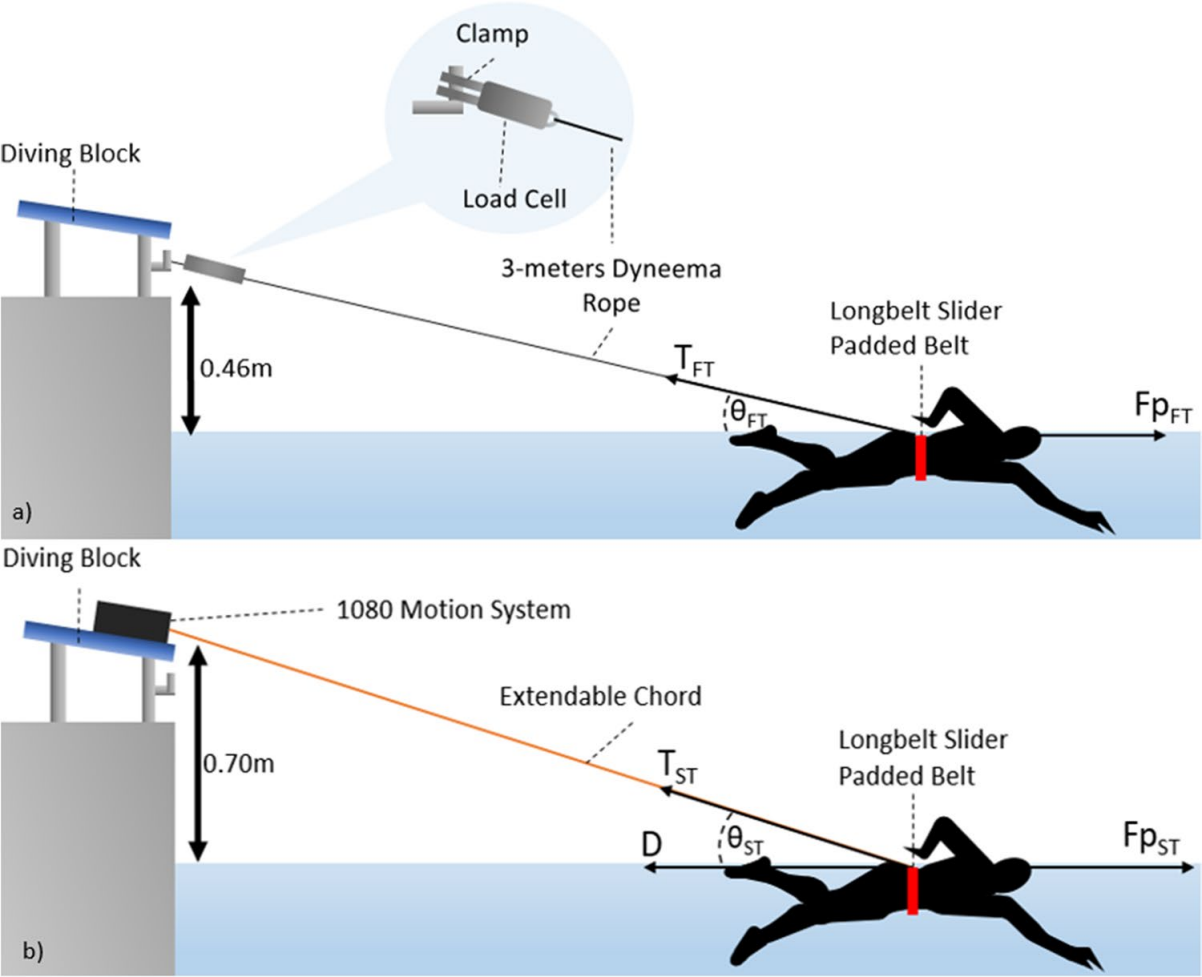
(**a**) Fully-tethered tension force equipment set-up and free body diagram. (**b**) Semi-tethered tension force equipment set-up and free body diagram^18^.

The fully-tethered set-up contained a DDEN-1000N-003-000 submersible load cell (Applied Measurements ltd, UK)^15^, attached to a competition diving block via a clamp. An accompanying DSCUSB Strain Gauge Digitiser and software allowed the equipment to measure applied load during the experiments at a sampling frequency of 100 Hz. A 3-m length of SK78 Dyneema Dinghy Rope (Marlow Ropes ltd, UK) was attached to a clamp (CPC, UK), with the other end attached to a Longbelt Slider Padded Belt (StrechCordz, New Zealand), fitted around a swimmer’s waist as shown in Fig. 1a. The load cell records tension force values of the rope caused by the swimmer at each timestep.

The semi-tethered set-up used a 1080 Sprint^16^ (1080 Motion, AB, Lindingo, Sweden) robotic resistance device to record velocity and force readings at a sampling frequency of 333 Hz for each applied external load produced by the athlete swimming. The 1080 Sprint records the tension force in the rope, distance, and velocity of the athlete at each timestep.

Finally, a set of SmartPaddles (Trainesense, Finland)^17^ were used exclusively as a tool to aid identification of stroke cycle, not to supply reaction force data for analysis. The paddles record the reaction force applied by the water on the hands at a sampling frequency of 40 Hz, allowing indication of when the stroke cycle begins and finishes based on the recorded reaction force values.

### Procedure—experiment

Following a self-selected 1000 m warm-up, experiments took place in a 50 m × 25 m swimming pool, with two data collection stations located at either end. To minimise the risk of order bias and fatigue, swimmers were randomly allocated to commence data collection at each station and performed a 600 m recovery swim between experiments.

Data was collected via the following two sub-experiments:

1. Fully-tethered force (no drag)
  Figure 1a displays the equipment set-up used in the fully-tethered tension force trials. Using the stationary load cell set-up, athletes would start the test in the water. Athletes were instructed to swim front crawl for 25 s at maximum effort, facing away from the load cell while remaining stationary. Athletes were instructed to rest for 5 min before repeating the experiment a second time. Athletes were instructed to breathe minimally throughout the experiment. The SmartPaddles were worn during this experiment due to the ease of starting and stopping the equipment, as indicated during the pilot testing.
2. Semi-tethered force (includes active drag)
  Figure 1b displays the equipment set-up used in the semi-tethered tension force trials. Using the 1080 Sprint, athletes started in the water, sculling slowly away from the 1080 Sprint until a calibration distance of 5 m was reached. Athletes would then swim front crawl with maximum effort until they passed the 25 m mark in the pool. Athletes were instructed to breathe minimally throughout the experiment. The minimum value of resistive force available on the 1080 Sprint was selected, equal to 9.81 N, to minimise the impact of the equipment on an individual’s technique. The athlete was then slowly towed back to the starting position with the experiment repeated after 5 min of rest.
  The experiment was videoed underwater using two GoPro Hero 9 Black cameras (GoPro Inc., USA) and overwater using two Canon Legria GF25 cameras (Canon Inc., Japan) to provide assistance in the post processing section of the experiment. No video footage images have been made viewable within the paper.

### Post processing

A number of assumptions were made during the experiment:

1. A consistent power output is used by the athletes during each part of the experiment, to ensure the force values found are comparable between different trials.
2. A consistent technique is used by athletes during each part of the experiment, to ensure comparison between different trials is accurate.
3. The 1080 Sprint, SmartPaddles and fully-tethered experiment will have limited impact on the overall technique of the athletes during the trials.
4. It was assumed the stroke rate of the fully-tethered and semi-tethered experiment was constant for each trial.

A consistent power output is an assumption used regularly in swimming and active drag literature, such as the VPM method^11^. A consistent technique is an assumption that must be made as to ensure the gait of the strokes of an individual athlete can be compared. The use of fully-tethered equipment and its limited impact on technique is a common assumption made in swimming and active drag literature^19^. The impact of the SmartPaddles on the athlete’s technique is limited, as displayed and discussed in the supplementary information section titled ‘SmartPaddle Impact Tests’. Also included in this section are results that show the limited impact of the SmartPaddles on the load cell used in the fully-tethered load cell experiment. Unlike the VPM, the wetted surface area of an athlete is not required, meaning the complex calculation for the wetted surface area of different body positions can be avoided^11^. Avoiding this assumption means errors are not introduced by inaccurate prediction of this wetted surface area. The erroneous assumption of a constant velocity across the whole stroke, as seen in the MAD system is also avoided^9^. By avoiding this constant velocity, an average value of active drag across the whole stroke cycle is avoided, allowing for the transient active drag profile across a stroke cycle to be estimated.

To account for the impact on results caused by the height of the equipment above the water, results were resolved into horizontal and vertical components with the horizontal component derived from the tension force in the rope produced by the swimmer. The trigonometric equations used to find this horizontal component of the tension force in the rope is shown in Eqs. (6 and 7). ${T}_{HFT}$ and ${T}_{HST}$ refer to the horizontal component of the raw tension force in the rope collected by the fully tethered load cell and 1080 Sprint, respectively (${T}_{FT}$ and ${T}_{ST}$), when the rope is at an angle. The angles ${\theta }_{FT}$ and ${\theta }_{ST}$ are the angles between the rope and the water of the fully tethered load cell and 1080 Sprint respectively. Gonjo and Olstad used a similar equation in their prediction of active drag, in order to account for the impact of the angle of the rope caused by the height of the equipment^20^.

6 $$0\le \theta \le 90 \quad {T}_{HFT}= {T}_{FT}\mathrm{cos}{\theta }_{FT}$$

7 $$0\le \theta \le 90 \quad {T}_{HST}= {T}_{ST}\mathrm{cos}{\theta }_{ST}$$

**Figure 2:**
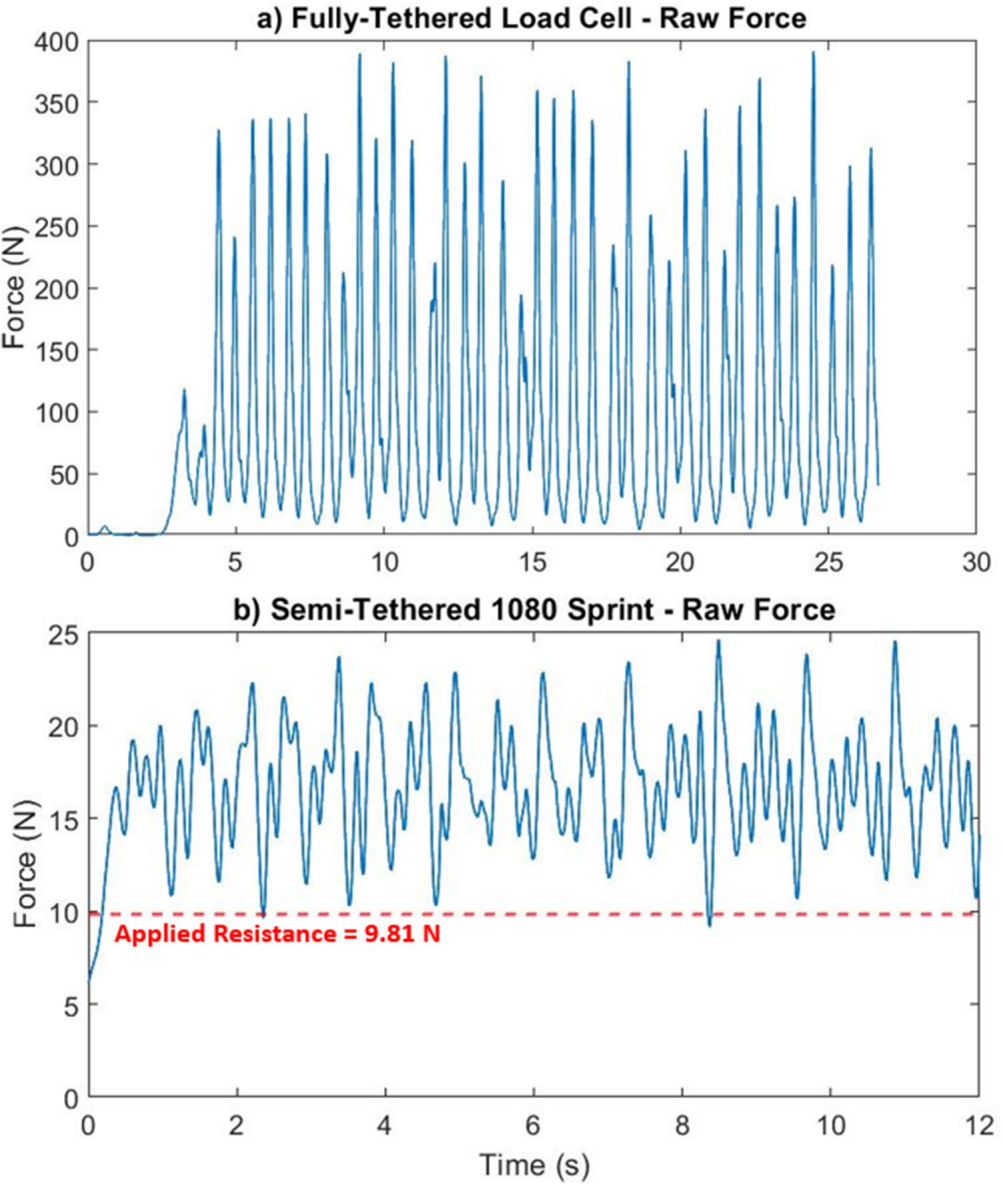
Raw tension force found via (**a**) fully-tethered equipment (**b**) semi-tethered equipment.

The next step involved identifying appropriate stroke cycles for analysis. Unsynchronised video footage of the athletes collected during the experiments aided in identifying different stroke cycles. In the raw data collected by each piece of equipment, graphs displayed a number of peaks and troughs as shown in Fig. 2a,b in the results section. These peaks and troughs help indicate when a stroke cycle changes, with approximately two peaks and three troughs indicating one full stroke cycle. To identify stroke cycles that could be used in analysis, the peak values of the resolved tension force data collected by the fully-tethered load cell and the 1080 Sprint were plotted using the findpeaks function in MATLAB’s signal processing toolbox^21^. A minimum peak value constraint was added to ensure no artificially low peaks were identified and mistaken for the maximum resolved tension force values produced in the stroke cycle. This minimum peak value was set equal to the average value of the resolved tension force data collected across the full experiment time by each piece of equipment.

A boxplot of peak resolved tension forces across the full stroke cycle was plotted, indicating which stroke cycles could be used for analysis. As indicated by the boxplot, any outlying peak resolved tension force values found resulted in the appropriate stroke cycle being excluded from further analysis. The method of evaluating appropriate stroke cycles for further analysis is described in the main experiment section of the supplementary information. As per standard boxplot conventions, potential outliers were identified as values more than 1.5 times the interquartile range above or below the 25th–75th percentile range of the main box. The start and end of the stroke cycles also had to be easily identifiable in order to be considered for further analysis. Stroke cycles located at the start/end points of the recorded data were also excluded from the analysis. Example boxplots with accompanying explanation of the stroke selection procedure have been included in Fig. 2 in the supplementary information.

It was found (via the data collected and provided by the SmartPaddles, accompanying software and video analysis) that the minimum force registered during a stroke cycle occurred approximately at the start/end point of the stroke. Based on this fact, the start/end points of each stroke cycle were assumed to occur at each minimum local resolved tension force value recorded by either the 1080 Sprint or fully-tethered load cell. Examples of these minima values can be seen in the results section in Fig. 2a,b at around 10 N for both the fully-tethered load cell and the 1080 Sprint system. The data of the first two strokes collected via the 1080 Sprint and the data from the first 4-s of the fully-tethered load cell set-up were also ignored to ensure data was not influenced by the start of each trial. Video footage of the trials aided in identifying if a stroke cycle began on the left or right arm, to ensure comparison was as consistent as possible throughout.

Seven strokes were manually selected in Microsoft Excel, based on the boxplots described above, from each set of collected force data to ensure the full trial was represented. To ensure the same point in the stroke was compared between experiments, time was non-dimensionalised with force data collected for each stroke resampled via MATLAB’s resample function. The resampling resulted in approx. 350 time based sample points being converted into 101 position based sample points, each accounting for force recorded at each 1% interval of a full stroke cycle. It was assumed the athlete swam with consistent technique, meaning the data collected for the same point in different trials could be compared.

Within the *fully-tethered* experiment, the load cell collected force values that represent the tension in the rope caused by the athlete swimming against the load cell. The load cell was zeroed before each trial to account for the weight of the rope in the collected force readings. As the athlete is stationary, it is assumed there is negligible drag force acting on the athlete while attached to the load cell. At each 1% position of the stroke cycle, it is assumed the system of forces is in static equilibrium and tension will be maintained on the rope as the athlete swims against the equipment.

The free body diagram of the fully-tethered experiment is included in Fig. 1a. During the fully-tethered experiments, the propulsive force acting on the swimmer is calculated as in Eq. (8):

8 $${Fp}_{FT}(t)= {T}_{HFT}(t)$$

Within the *semi-tethered* experiment, the 1080 Sprint collected force values that represent the tension in the rope caused by the athlete swimming away from the equipment. The maximum weight of the rope when extended to 25 m, approximately 100 g, will have a negligible impact on the force recorded by the 1080 Sprint and has therefore been assumed to be zero. As the athlete is moving through the water, there is a drag force acting on the athlete dependent on the velocity at which the athlete is swimming. At each 1% position of the stroke cycle, it is assumed the system of forces is in dynamic equilibrium and tension will be maintained on the rope as the athlete swims away from the equipment.

The free body diagram of the semi-tethered experiment is included in Fig. 1b. During the semi-tethered experiments, the propulsive force acting on the swimmer is calculated as in Eq. (9):

9 $${Fp}_{ST}(t)= {T}_{HST}(t)+D(t)$$

With the assumption that ${Fp}_{FT}$ is equal to ${Fp}_{ST}$, the active drag, ${F}_{DA}$, can then be calculated by combining Eqs. (8 and 9) into Eq. (10).

10 $${F}_{DA}(t)= {T}_{HFT}(t)- {T}_{HST}(t)$$

Following this method at each 1% position in the stroke cycle will result in the active drag value being calculated at each position during the stroke cycle, allowing the active drag profile to be displayed across the full stroke cycle. This process was conducted using the corresponding sampled strokes on both pieces of equipment, i.e. data from sample stroke 1 of the semi-tethered equipment was subtracted from data from sample stroke 1 of the fully-tethered equipment.

## Results

The raw results displayed in Figs. 2 and 4 correspond to one of the fourteen trials conducted, unless otherwise stated. Figure 2 displays raw data collected from the fully-tethered and semi-tethered equipment.

The fully-tethered load cell set-up experiment shows a maximum force of around 400 N, with the majority of peak forces having values between 250 and 350 N. The 1080 Sprint recorded peak force values of around 25 N, with the majority of results between 10 and 25 N.

**Figure 3:**
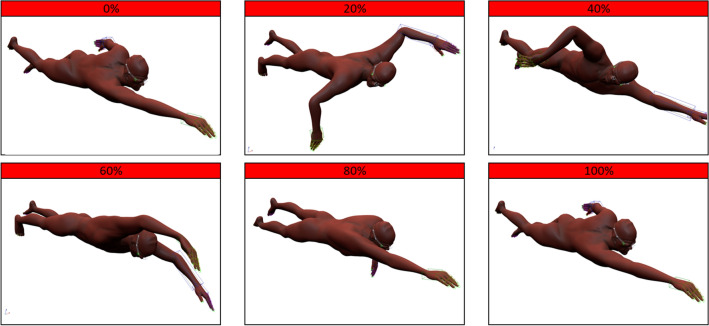
Corresponding front crawl positions to appropriate stroke cycle percentages^18, 22^.

In order to aid understanding of the horizontal axis on the following active drag graphs, a stroke cycle percentage diagram composed of a swimming geometry and animation designed by CodeThisLab^22^ was included (Fig. 3). A single stroke cycle was extracted from the animation and broken down into six positions based on percentage of stroke cycle, with the 0, 20, 40, 60, 80 and 100% of timestep positions included and shown in Fig. 3. The images of the animation stroke cycle percentage points were taken in 3D Studio Max. These images approximately correspond to the positions of the athlete, at the corresponding stroke cycle percentage values, throughout the graphs in the results section, although the positions are not exact and will vary between each athlete’s technique.

**Figure 4:**
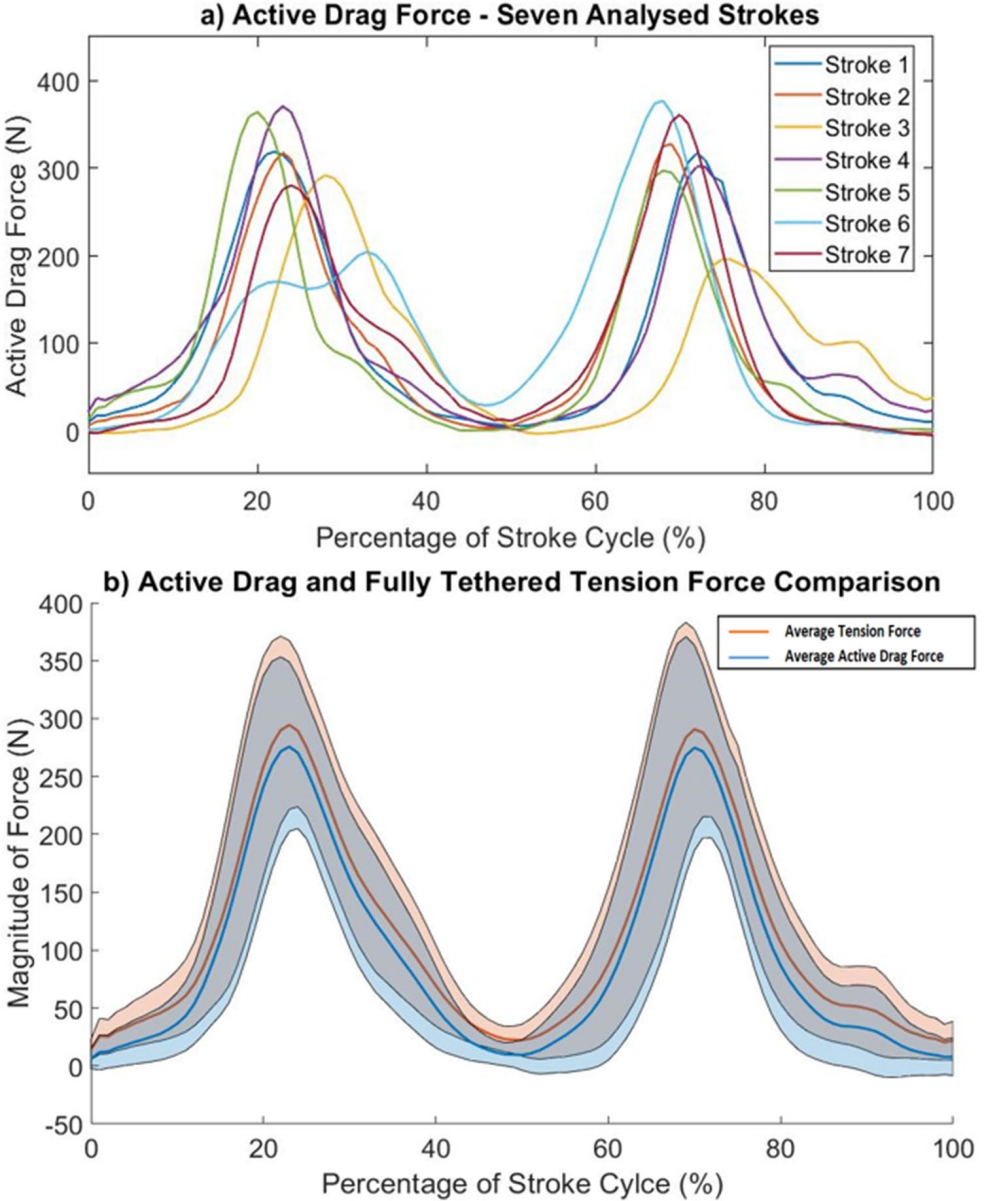
(**a**) Individual active drag values across seven full stroke cycles during one trial. (**b**) Average active drag plotted alongside propulsive force magnitude, with standard deviation of each force indicated by shaded regions around the average curves.

The active drag values displayed in the following graphs were calculated for seven full stroke cycles, selected as described in the boxplot discussion above in the main text and supplementary information, for one trial. Although plotted based on percentage of stroke cycle, the results implicitly show the change in active drag with time as the position of the athlete is a result of the time spent taking a stroke. Figure 4 shows how the active drag value changes over the stroke cycle percentage, corresponding to the positions in Fig. 3.

Figure 4a shows the maximum active drag forces on a stroke cycle vary from around 300 N to 375 N with the minimum active drag values in the stroke cycle being close to 0N. Figure 4a also indicates that an athlete’s active drag force can vary greatly between different stroke cycles, varying by approx. 100 N around the peaks. Figure 4b shows the average active drag values of the seven sampled stroke cycles, shown in Fig. 4a, plotted on the same graph as the average fully-tethered propulsive force magnitude for the same seven sampled strokes, as measured during the fully-tethered experiment. Also included are the values of standard deviation for the propulsive and active drag forces, displayed as shaded regions around the average propulsive and active drag curves.

Figure 4b shows the difference between the active drag force and the propulsive force magnitude varies across the cycle between around 10 N and 30 N. Based on Newton’s first law, the tension force recorded by the load cell during the fully-tethered experiment should be fluctuating around and approximately equal to the calculated active drag force, indicative of the acceleration and deceleration within the stroke cycle, although this is not the case. The difference, amounting to between 10 and 30 N depending on position, is caused by the tension of the rope in the semi-tethered experiment. As the athlete is attached to the 1080 Sprint while moving through the water, the tension present in the rope will act to slow down the swimmer to a velocity that is artificially lower than during free swimming. This lower velocity means the active drag acting on the swimmer is lower during semi-tethered swimming than during free swimming, although the propulsive force in both cases is assumed to match.

**Figure 5:**
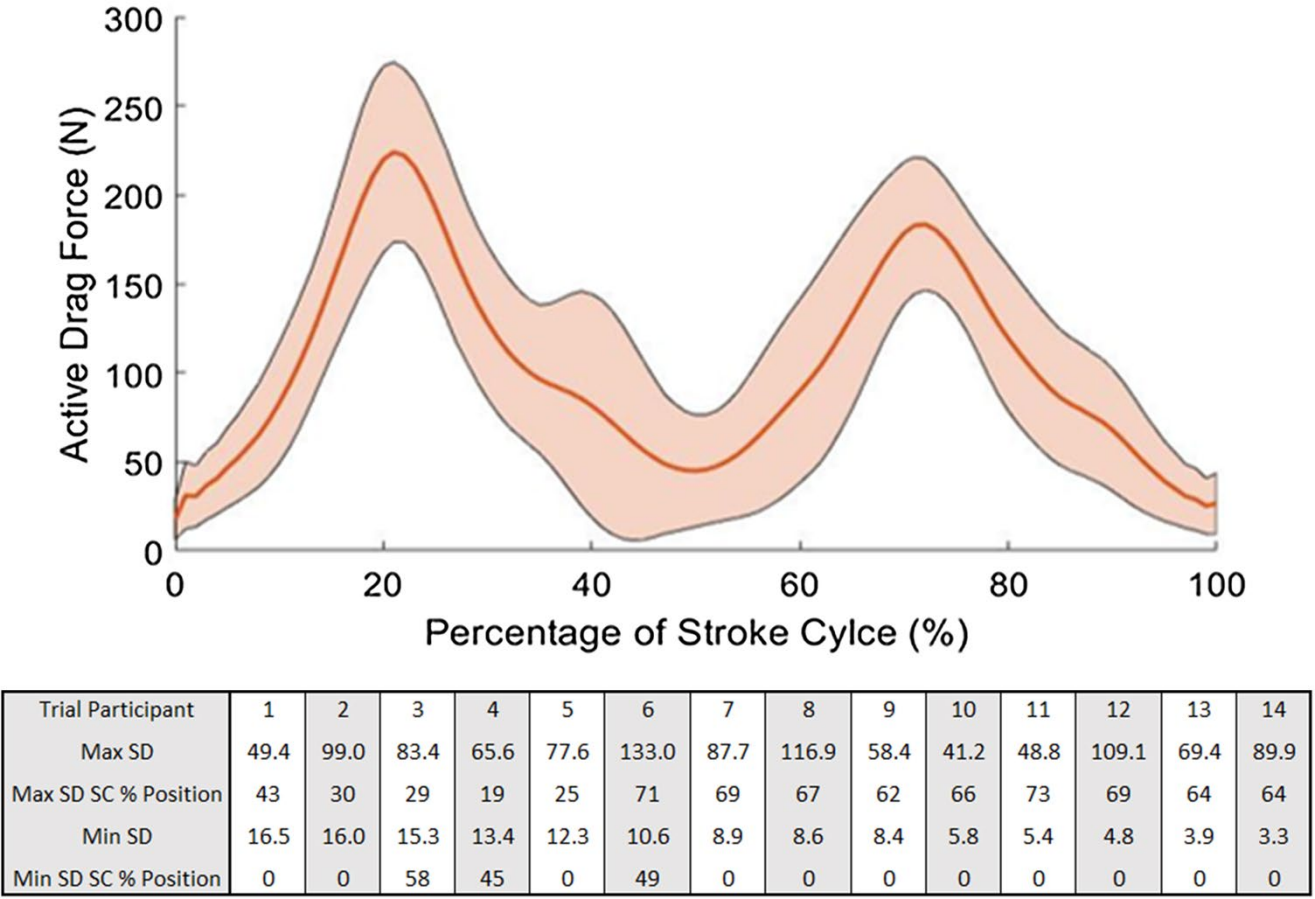
(**a**) Active drag plotted as a percentage of stroke cycle for fourteen averaged trials with standard deviation included (shaded region). (**b**) Table showing the maximum and minimum standard deviations for each trial.

Figure 5 displays the averaged active drag results of seven sampled strokes on each of the 14 trials.

Figure 5a shows the maximum active drag forces peak between 200 and 250 N, with the maximum standard deviation being 63.9 N occurring at 41% of the stroke cycle. The high values of standard deviation show the large variation possible of the active drag at similar points in the stroke cycle, when comparing different athlete’s techniques. The maximum and minimum values of standard deviation for each trial are included in Fig. 5b, showing the range of standard deviation values within the stroke cycle across an athlete’s 7 strokes used in the analysis. Further results of this experimental method are discussed in the ‘Pilot Testing’ section of the supplementary information, detailing the improvements made to the experiments based on the results found from the pilot testing sessions.

## Discussion and further analysis

### Active drag profile

The aim of this experiment was to propose a method of measuring the transient active drag of an athlete over a full stroke cycle, avoiding the need for complex calculations based on approximate values. Existing methods of active drag measurement provide a range of individual active drag values at various swimming velocities.

Figure 3 assists Fig. 4 in displaying the values and locations of maximum and minimum drag, with minimum drag values occurring when the swimmer is in a more streamline position with one arm stretched out in front. Maximum drag values occur during the catch and pull phase of the stroke cycle when the body is in a less streamlined position. Figure 4 does show areas of active drag with results close to 0 N, but this is unlikely to be the case as the drag of an object cannot be zero when moving at a velocity greater than 0 ms^−1^ in a fluid at ambient temperature; i.e. zero drag can only occur in a vacuum. Results close to 0 N could be as a result of the subtraction of data described in Eq. (10), depending on the magnitude of the fully-tethered and semi-tethered data used in the subtraction.

During the fully tethered experiment, there is always a positive value of tension recorded by the load cell as the athlete attempts to produce a propulsive force across the full stroke cycle. As the body is stationary, the system will remain in static equilibrium, meaning the horizontal and vertical forces acting on the body are balanced. The drag force acting globally on the stationary body is negligible, with all resistance coming from the reaction force of the rope at the wall. The minima of the force curves, shown in Fig. 4, display the locations within the stroke cycle that small amounts of propulsive force are generated, potentially caused by the leg kick alone or the transient nature of the experiment, although this would need further investigation. Because of these small values of propulsive force being produced and the absence of drag acting on the body, tension was maintained on the rope, as indicated by the raw results in Fig. 2.

From Fig. 2b, it can be seen that most of the local minima do not fall below 9.81 N, as indicated by the red dashed line in Fig. 2b. This 9.81 N value is significant as it is equal to the pre-set resistive force applied to the 1080 Sprint. As the points of the curve never fall to 0 N, this shows that the equipment acts to maintain a resistive force of 9.81 N on the rope across the full semi-tethered experiment. It can be seen that two of the minima fall below the resistance force of 9.81 N, likely caused by equipment lag in maintaining a constant resistive force. The minima that fall below the value of 9.81 N would likely have been portions of the experiment that experienced rope slackening, had it been possible to set no resistive force for the equipment. The positive value of force measured across the experiment therefore implies that tension is being maintained on the rope.

The mean active drag across the full stroke cycle was calculated for each of the 14 completed trials. The data showed the mean active drag for a full stroke cycle ranged from 76 to 140 N, depending on the trial. It was found that 12 out of 14 trials were in the range of 94 N to 140 N. The mean swimming velocity (obtained from the 1080 Sprint) found experimentally was 1.73 ms^−1^. The peak values of active drag ranged between 175 and 300 N across the 14 trials, at the approximate corresponding stroke cycle positions of 20% and 70%.

Direct comparison to the results of Gonjo and Olstad^19^, who also used a 1080 Sprint but adopted the VPM method to measure a mean drag of between 46 to 84 N in males at a velocity of approximately 1.82 ms^−1^ for resistive loads ranging from 1 to 9 kg, shows similar results in terms of magnitude, with results collected using the methodology proposed in this study 30–50 N higher than those collected by Gonjo and Olstad. Based on the drag equation included in the work of Webb and Banks^8, 9^, the value of active drag found by Gonjo and Olstad should be greater than the active drag estimated using the methodology proposed in this study, due to a higher value of average velocity. A reason for the difference in results could be the calibre of participants used by Gonjo and Olstad being higher than those used in the current experiment. A higher calibre of athlete would imply a better skill set with less associated active drag. The calibre of the group of athletes used in the current study is likely lower than those used by Gonjo and Olstad, based on FINA points comparison. The *highest* FINA point score amongst the swimmers participating in this study was approx. 690, which approximately equalled the *average* FINA Point score of the athletes in Gonjo and Olstad’s study^19^. Another reason for the difference in results between the two methods could be the adopted VPM approach used by Gonjo and Olstad being sensitive to experimental error, as stated in a literature review by Formosa et al.^8^.

Kolmogorov and Duplishcheva^23^, using the VPM, found mean active drag results of between 42 and 167 N at velocities of between 1.5 and 1.9 ms^−1^, directly comparable with the method used in this experiment. Mason^24^, using an assisted towing method similar to the NABA method, approximated mean active drag values of between 112 and 253 N at a mean max swimming velocity of 1.82 ms^−1^. Average active drag values at the lower end of Mason’s study, 112 N, are comparable with the average active drag results collected by the methodology outlined in this study. Mean active drag values at the higher end of Mason’s study, 253 N as stated in the literature, are comparable with the peak active drag values calculated in the current study. The largest peak active drag values, considering all 14 trials, of approximately 175–300 N correspond to the least streamlined positions, as shown in Fig. 4 in the 22% and 72% positions.

Although this does compare a mean value across a stroke cycle, conducted by Mason, to a mean value at a certain point in a stroke cycle, the magnitudes are similar, indicating the current method is accurate in terms of magnitude of results. Mason made use of a flux vector dynamometer mounted on a Kistler force platform in order to tow the swimmers and measure the towing force required, using a tachometer to validate the velocity of towing^25^. Mason adopted a VPM, meaning one reason for the differences in average active drag results could be due to the sensitivity to experimental error. Mason also towed the swimmers toward the towing rig at artificially increased velocities, which could have affected and inflated the active drag value.

### Analysis of potential for improving insight into swimming technique

One of the motivating factors for measuring the transient nature of active drag is to enable deeper analysis of the link between active drag and other swimming metrics. As an example of possible usage, further analysis was conducted on the active drag profiles of the 14 trials considering the stroke rate of each athlete. The values of stroke rate for each trial where calculated based on videos taken during the fully-tethered experiment, using the Stroke Rate Stopwatch iPhone application^26^. The app allows manual approximation of the stroke rate of a series of strokes before averaging the stroke rate across the total number of strokes considered. The stroke rate was taken twice, based upon 10 strokes around the midpoint of each fully-tethered experiment, and averaged. Due to camera position, the stroke rate could not be approximated during the semi-tethered experiment meaning the stroke rate was assumed constant across the experiments in each trial.

A full width half max (FWHM) analysis of a full stroke cycle, indicating the percentage range of stroke cycle positions that experience high values of active drag compared to the minimum value of active drag of the stroke cycle, was used to investigate a potential link between stroke rate and active drag. The FWHM value was found for the two existing peaks in each of the 14 active drag trial force curves, with both FWHM values added together to find a total FWHM value across the full stroke cycle for each separate trial. Similar to the identification of peak values in the methodology section, this was completed using the findpeaks function in the signal processing toolbox in MATLAB.

**Figure 6:**
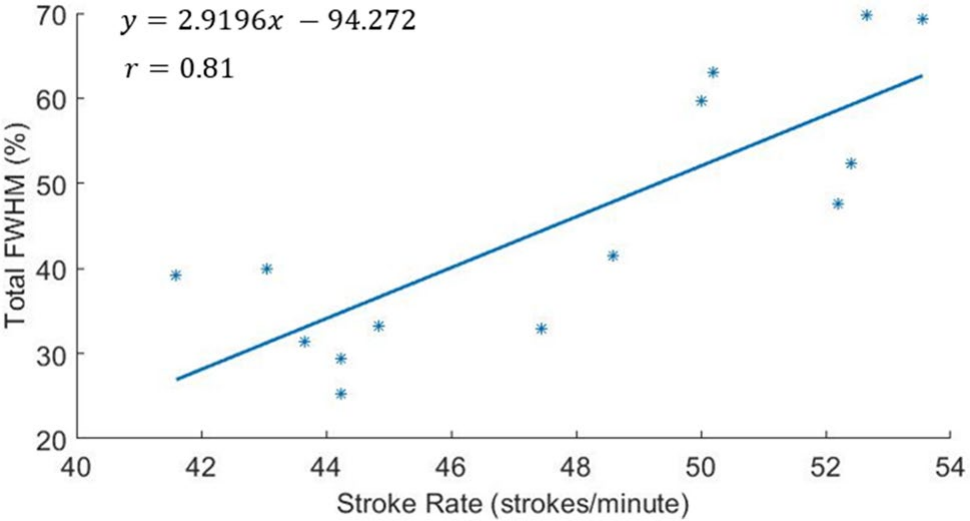
Full width half max values of active drag profiles plotted against the stroke rate for each of the 14 trials.

The results clearly show that as the stroke rate increases, so too does the portion of the stroke cycle spent at higher values of active drag (Fig. 6). The correlation coefficient value ($r$) between stroke rate and FWHM value is equal to 0.81. Other approaches for estimating active drag reviewed earlier cannot currently verify this relationship as they do not include the full transient active drag profile. Thus, this type of analysis demonstrates a potential benefit of the current proposed method relative to others.

One observation during the FWHM analysis was that at higher values of stroke rate, the peaks of the active drag curve became more challenging to identify, as the drag value remains higher for a larger portion of the stroke. As a result, it is possible that the FWHM value was overestimated at these higher stroke rates. This is not the case at lower stroke rates. If the most conservative approximation of FWHM is made for each trial, correlation was still high ($r=$ 0.63). These results imply that an ideal active drag profile would likely include two sharp and narrow peaks, peaking at high values of active drag, indicating a large propulsive force being applied for a short period, preventing drag from acting over an increased stroke duration. While this suggests that there could be an optimum stroke rate for an athlete’s front crawl technique, this would need further investigation through further trials and is beyond the scope of the current study.

### Experimental repeatability

From Fig. 5a, the high values of standard deviation indicate there is a large variation in active drag between different trials, stemming from the raw data collected by the equipment. Figure 5b shows a comparison of the standard deviation results for each of the 14 trials. The standard deviation was taken across the seven sampled strokes at each percentage point of the stroke cycle.

The data in Table 2 shows that there is a large range of maximum standard deviation, with the average maximum standard deviation being 80.7 N. The minimum value of standard deviation varies considerably less between athletes, with the average minimum standard deviation being equal to 9.5 N. No correlation was found between the stroke rate of a given athlete when compared to their maximum and minimum standard deviations or between the total FWHM value of the athlete’s stroke cycle and their maximum and minimum standard deviations. It should be noted that in 93% of cases the maximum values of standard deviation occurred between the FWHM limits of the mean active drag curve produced when all 14 trials were considered. This implies that the least repeatable portion of the stroke cycle is approximately in line with the peak active drag positions, i.e. between 15–30% and 65–80% of the stroke cycle positions. Although the reason for the link between peak active drag positions and locations of minimum repeatability would need further investigation, results could be reported to coaching staff to indicate areas in which the repeatability of an athlete’s technique could be improved. In 100% of cases, the location of the minimum standard deviation occurs outside the region of FWHM (15–30% and 65–80%) with the most common location at the starting position of the stroke cycle, i.e. 0%. This could be due to the fact that an athlete can attain this relatively simplistic position more easily, meaning repeatability is likely to be higher due to potential for less variability in technique and more consistency in position. Again, the cause of any link between minimal active drag positions and locations of maximum repeatability would need further investigation, as they are beyond the scope of this study.

**Figure 7:**
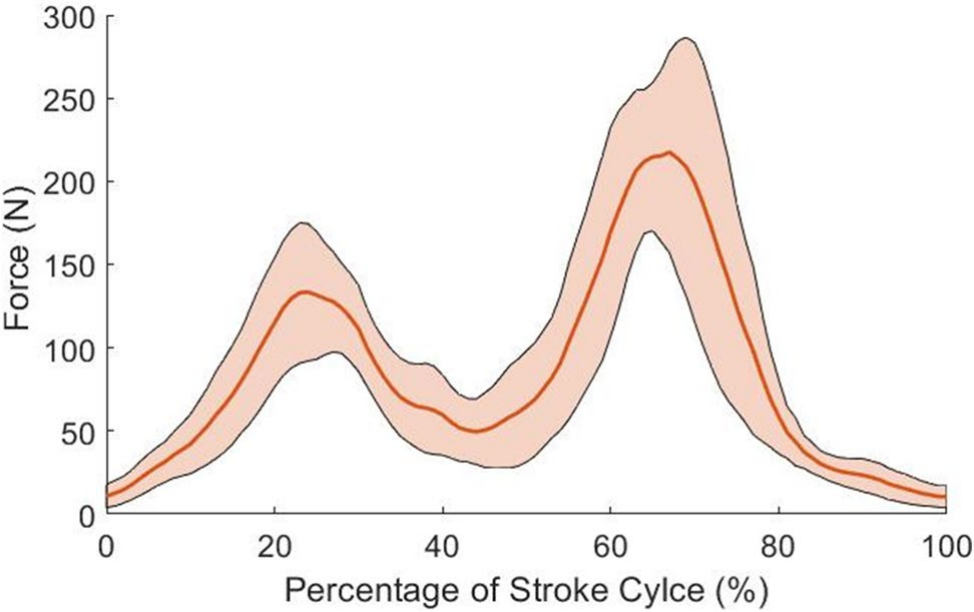
Propulsive force plotted as a percentage of stroke cycle for an individual completing six trials with standard deviation included (shaded region).

To further investigate the variation in collected raw tethered force values of *individual* athletes, the fully-tethered tension force experiment was repeated using a single athlete with a slight variation in methodology. The athlete completed a 25 s maximum effort front crawl swim while attached to the equipment, followed by a rest period of 5 min. The test was repeated a further five times. From each 25 s test, three concurrent strokes were sampled for analysis, with 18 strokes being analysed in total. Similarly, time was non-dimensionalised with each stroke cycle resampled into 101 points, each accounting for 1% of a full stroke cycle. The results show in greater detail how the raw tension force can vary for one athlete, with Fig. 7 showing the variation in raw tension force results collected by the fully-tethered load cell set-up.

The fully-tethered load cell set-up experiment shows a maximum standard deviation of 88.3 N occurring at 71% of a stroke cycle. Once again, this maximum standard deviation has been produced at values within the FWHM range, similar to what was observed above in Fig. 5b. The minimum value of standard deviation is outside the range of values associated with FWHM, as noted above in Fig. 5b. These results imply that an individual’s repeatability of power output is limited during maximum effort swimming, with variation likely between each stroke cycle. The high values of standard deviation are likely due to changes in an individual’s propulsive power output, technical changes between strokes, breathing patterns and fatigue, although further investigation would be required.

Based on consideration of the limitations and results discussed above, some recommendations on improving accuracy and repeatability of the method can be recommended for future work. A synchronised camera set up on all experiments would mitigate the manual approximation of the start/end of the stroke cycle, ensuring repeatable comparison was more likely. The variation of an individual’s results between strokes and trials indicates mean experimental results should be used during post processing in order to approximate an individual’s average active drag. In order to do this for each person, more repetitions would be required on each piece of equipment, similar to the methodology used in the fully tethered repeatability experiment.

## Conclusions

In conclusion, the conducted experiment details a method by which the active drag profile can be found across the full stroke cycle, while under some small resistance to forward motion. Calculated values of active drag are agreeable with values found by various existing experimental means, although comparison of the velocities responsible for the drag between experiments would need further investigation. Using this method, the active drag profile can be used to investigate the relationship between stroke rate and time spent at high values of active drag. Experimental accuracy could be improved using synchronised video footage and increasing the trials conducted by each athlete, in order to improve mean propulsion estimates. Synchronised video footage would also aid in accurately displaying the positions of the athlete at each corresponding stroke cycle percentage point. The reliability of this method, although agreeable with other active drag experiments, should be further investigated. In future, computational fluid dynamics (CFD) simulation will use the present data as a source of validation.

## Supplementary Information

Supplementary Information.

## Data Availability

Anonymised datasets used during analysis are available from one of the named authors, Alex Lennon, on reasonable request. Video footage of the experiments will not be made available at any time, in accordance with ethics approval.

## Abbreviations

2D: 2 Dimensional
3D: 3 Dimensional
CFD: Computational fluid dynamics

## VPM

${F}_{r1}$: Active drag resistance force
${F}_{b}$: Resistance force of towed object
${V}_{1}$: Velocity without towed object
${V}_{2}$: Velocity with towed object

## NABA

R_Active_: Active resistance
R_Naked_: Naked hull resistance
R_Correction_: Correction factor
R: Hull resistance
T: Propeller thrust
J: Advance ratio
V: Advance velocity
N: Number of propeller revolutions per second

## Method

${Fp}_{FT}$: Propulsive force tethered experiment
${Fp}_{ST}$: Propulsive force semi-tethered experiment
*T*_*FT*_: Tension force tethered experiment
*T*_*ST*_: Tension force semi-tethered experiment
*D*: Active drag
