# Supplementary material for: A novel method of determining the active drag profile in swimming via data manipulation of multiple tension force collection methods
Source: Sci Rep. 2023 Jul 5;13:10896. doi: 10.1038/s41598-023-37595-y (PMC10322921; doi:10.1038/s41598-023-37595-y)
Supplement: Supplementary file 1 — Supplementary Information. [file 41598_2023_37595_MOESM1_ESM.pdf]

A novel method of determining the active drag profile in swimming via data manipulation of multiple tension force collection methods – Supplementary Information

A. Haskins[1*], C. McCabe[2], R. Kennedy[2], R. McWade[1], A.B. Lennon[1], D. Chandar[1]

[1]School of Mechanical and Aerospace Engineering, Queen's University Belfast, Belfast, BT9 5AH, UK.
ahaskins02@qub.ac.uk
d.chandar@qub.ac.uk
a.lennon@qub.ac.uk
rmcwade01@qub.ac.uk
[2]School of Sport, Ulster University, Belfast, BT15 1AP, UK.
c.mccabe@ulster.ac.uk
r.kennedy@ulster.ac.uk

This supplementary information presents experimental results and information that support the work conducted in the main report. The information has been included to demonstrate the full scale of the research, as well as highlight a number of considerations made during the course of the study. Full experimental details have not been included as some of this work is likely to be considered for further publication.

All the discussed trials in the supplementary information were approved by the local Queen's University Belfast ethics committee, namely the Engineering and Physical Sciences Faculty Research Ethics Committee, and were performed in accordance with the guidelines and regulations of the Declaration of Helsinki. All participants were provided with verbal and written explanations of the purpose, procedure, and risks related to the study and provided written consent. Informed consent was obtained from participants of the study.

1. Pilot Testing

The Pilot testing was conducted following a similar approach as described in the main 'methodology' section. The pilot tests were conducted on three separate days, investigating each of the following pieces of equipment respectively.

Pilot Test 1: SmartPaddles

Pilot Test 2: 1080 Sprint (Semi-tethered experiment) (Passive drag tow test also conducted)

Pilot Test 3: Fully-tethered load cell (SmartPaddles trialled at same time)

The pilot tests were conducted using two senior male swimmers from a local swimming club, both of whom were over 18 years of age. Each pilot test was repeated twice by the athletes, similarly to the methodology of the main experiments. This resulted in four pilot test trials. The active drag was calculated, as described in the 'methodology post-processing' section of the main experiments. From the pilot tests, it was found that the SmartPaddles did not aid in predicting active drag, but could prove beneficial in aiding stroke cycle identification.

In pilot test 1, the athletes performed a front-crawl swim for 25 meters at maximum effort, whilst wearing the SmartPaddle sensors. The athletes rested for 5 minutes between both repetitions. In pilot test 2, the athletes performed a front-crawl swim for 25 meters at maximum effort while attached to the 1080 Sprint. The athletes rested for 5 minutes between both repetitions.

A passive drag tow test was completed during pilot test 2. The passive drag tow test consisted of each athlete being towed for 25 meters in a streamline position, followed by 2 minutes rest between each repetition. The athletes were towed at an average velocity, equal to the average swimming velocity found during the two semi-tethered pilot test trials.

In pilot test 3, the athletes performed a front-crawl swim for 30 seconds at maximum effort while attached to the fully-tethered load cell. SmartPaddles were also worn during this trial. The athletes rested for 5 minutes between each repetition.

The active drag values peaked at approximately 200-250N, with the maximum standard deviation equal to 144N at 30% of the full stroke cycle (Figure 1). From the pilot tests, a number of key improvements for the main experiment were identified. The results indicated that considering more than three strokes per trial for analysis could provide a more accurate estimation of the active drag profile experienced during each trial. The importance of collecting clear video footage of the trials was highlighted, due to difficulties in stroke cycle identification during post processing. Pilot tests also suggested that, in order to reduce order bias on the equipment, swimmers should be split into groups with one group performing the full-tethered experiment first and one group performing the semi-tethered experiment first.

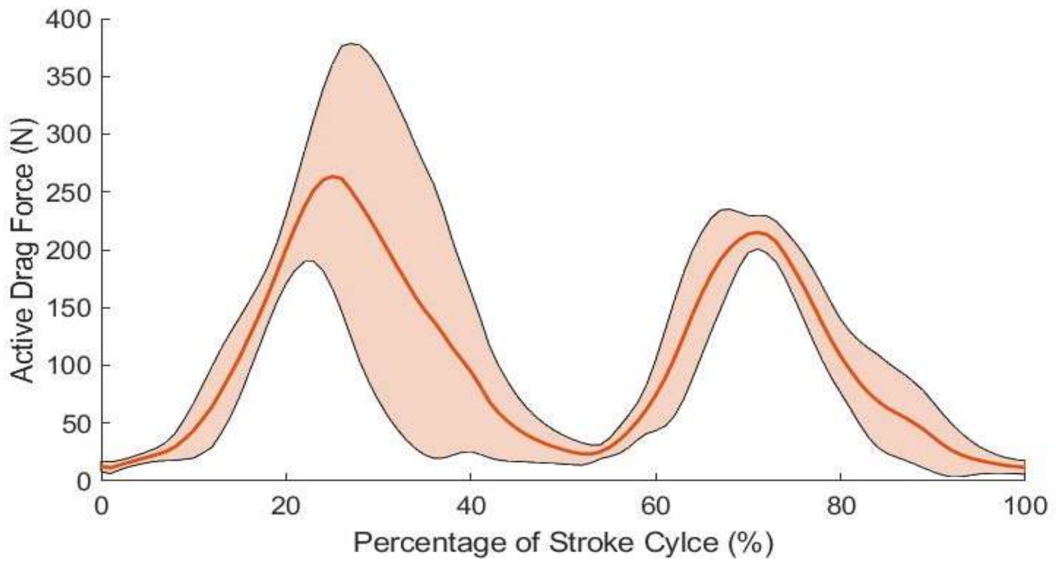

Figure 1: Mean active drag and standard deviation (shaded region) across all pilot test active drag trials.

Passive drag tow tests were planned for the main experiments, with the idea of trying to find a link between an athletes passive drag and active drag, potentially identifying the impact of passive drag on the final total of active drag. This relationship could not be found from the pilot test results or the main trial results and has not been included in the report.

2. Main Testing

Included in the following section are a sample of the boxplots used to help identify the peak values of collected raw data, as described in the main methodology. (Figure 2).

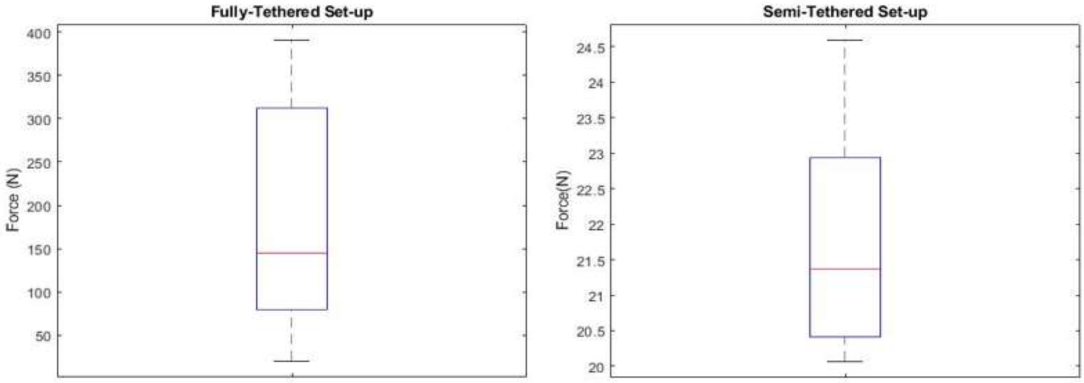

Figure 2: Force values collected via the SmartPaddles, which aided in stroke identification during post processing.

The SmartPaddles data can identify the time at which a new stroke is taken and the corresponding arm that takes the stroke, aiding to identify the approximate start and end of full stroke cycles (Figure 3). Although the SmartPaddles did aid somewhat as a stroke cycle identification tool, they were not used to determine the overall propulsive force acting on the body.

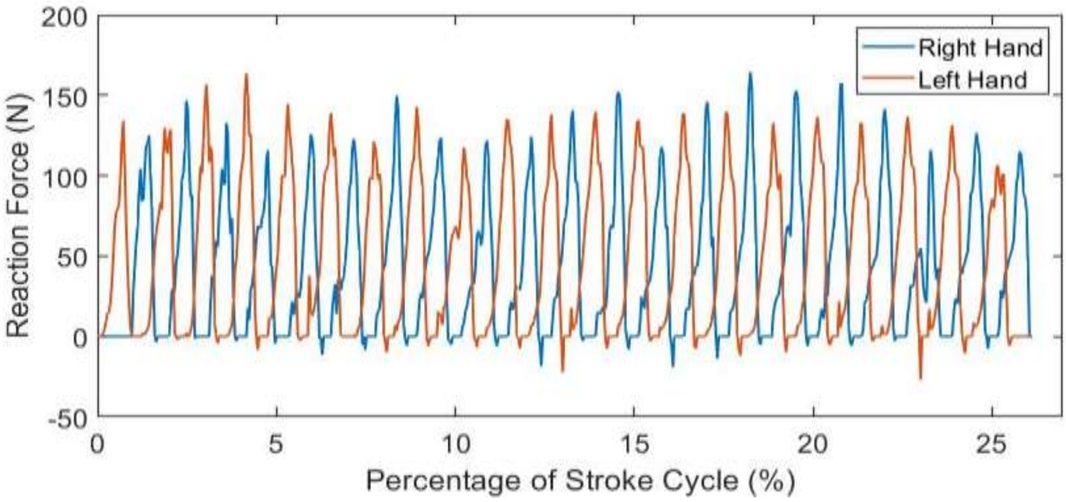

Figure 3: Example of reaction force profile for each hand during one of the front-crawl experiments using the SmartPaddles.

In order to find the stroke cycles deemed appropriate for further analysis, the boxplots shown in Figure 2 were used. The consistency of the stroke cycles, as displayed in Figure 3, indicated that using a boxplot to identify outliers for exclusion from further analysis would be appropriate. The findpeaks function from MATLAB's signal processing toolbox was used, as described in the main text, in order to identify the peak values recorded during each stroke cycle. A minimum peak height was added as described in the main text. As per standard boxplot conventions, potential outliers were identified as values more than 1.5 times the interquartile range above or below the 25th-75th percentile range of the main box.

3. SmartPaddle Impact Tests

Further trials were subsequently conducted investigating the impact of the SmartPaddles on the results of the fully tethered load cell. The experiment also investigated the difference in propulsive force results, collected by the SmartPaddles, between free and tethered swimming. The SmartPaddle Impact (SPI) experiment was conducted over three separate days, similar to the pilot testing, as follows:

SPI Test 1: SmartPaddles

SPI Test 2: Fully tethered load cell

SPI Test 3: Fully tethered load cell and SmartPaddles trialled in conjunction

A series of 12 trials were conducted using 6 athletes, all of whom over 16 years of age. Each of the three SPI tests were conducted twice by each athlete. In SPI test 1, the athletes performed a front-crawl swim (25 meters of maximum effort), resting 5 minutes between each repetition. In SPI tests 2 and 3, the athletes performed 30 seconds of maximum effort swimming while attached to the fully-tethered load cell. The athletes rested 5 minutes between trials. In SPI test 2, the athletes did not wear the SmartPaddles, whereas in SPI test 3, the athletes were instructed to wear the SmartPaddles. Due to injury concerns and participant availability, only 7 trials could be used for post processing and analysis.

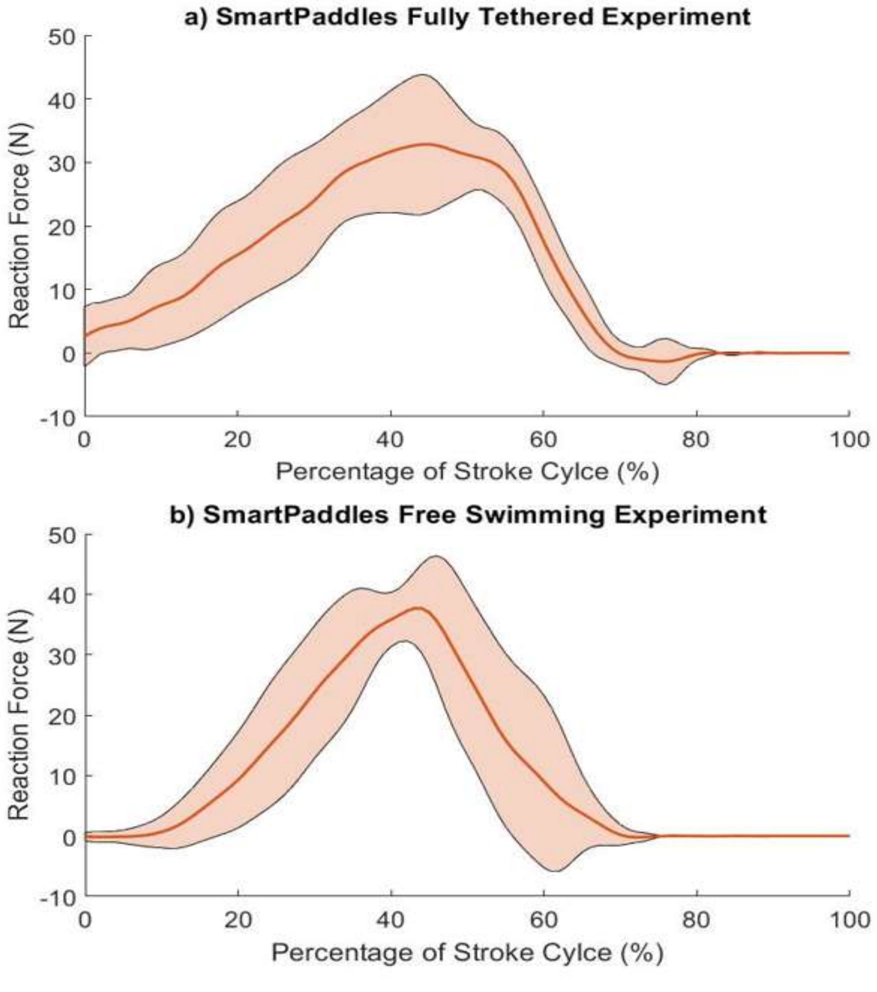

Figure 4: SmartPaddle propulsive force results during a) tethered and b) free swimming. The standard deviation is displayed as a shaded area around the average propulsive force curve.

The results show the average propulsive force profile and standard deviation for three full stroke cycles looking at the athlete's right hands for both tethered and free swimming (Figures 4a and 4b). The magnitudes and profiles of the propulsive force magnitude in both graphs are largely very similar between corresponding stroke cycle positions across the full stroke profile, with both graphs showing average curves peaking between 30 - 40N. One notable difference is the slightly stretched profile of the mean propulsive force curve, found during the fully-tethered trial, compared to the narrower profile of the propulsive force curve, found during free swimming. The FWHM values were calculated for both the fully tethered and free swimming SmartPaddle experiments, equal to 39.1 and 26.6 respectively. This shows that high values of reaction force are being produced for a larger percentage of the stroke cycle, whilst attached to the fully tethered experimental set-up than during free swimming. This could be due to subconscious bias caused by the fully tethered equipment, encouraging the athlete to attempt to produce more force, although this would need further investigation. The maximum standard deviation in both the free and fully-tethered cases are 11 N and 14 N respectively. These results confirm that there is a limited difference between how the forces act on the hand when free swimming and fully tethered swimming, supporting the methodology used in the study.

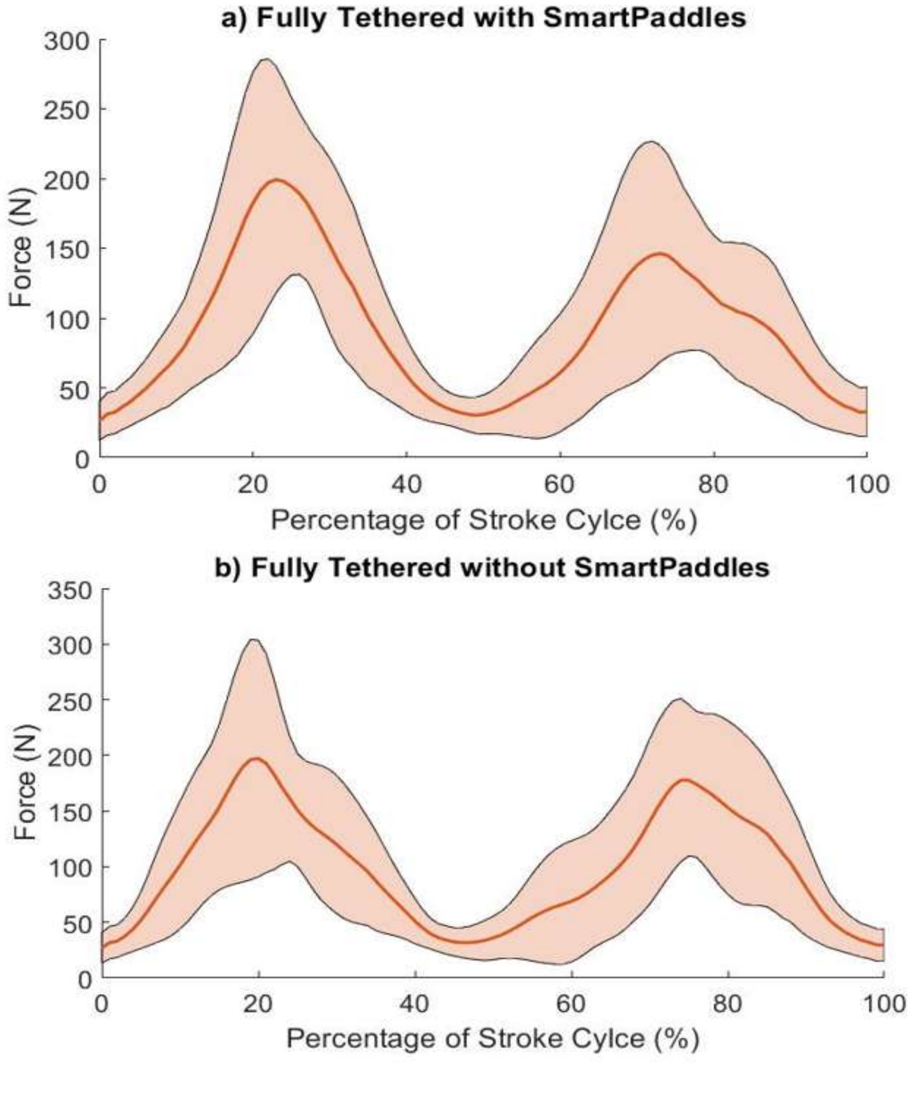

Figure 5: Fully-tethered load cell tension force results a) with and b) without the SmartPaddles. The standard deviation is shown as a shaded area in both Figures 5a and 5b.

The results of fully tethered swimming with and without SmartPaddles show average tension force profile and standard deviation for three full stroke cycles were similar (Figures 5a and 5b). Additionally, peak force values are relatively consistent between trials, with the profiles of the mean tension force curves following a similar trend on both graphs. The first peaks show a consistent value of around 200N between both graphs, although there is a discrepancy of 40N on the second peak. It is possible the SmartPaddles cause this reduced load on the tension force results, although it is likely this reduced load would be seen on both peaks if this was the case. The maximum standard deviations are approximately 107 N with no SmartPaddles and 93 N with SmartPaddles, showing that variation is relatively consistent between both experiments. The maximum standard deviation occurs at around 20% of the full stroke cycle in both experiments, again showing consistency between both sets of results. This evidence points towards the SmartPaddles, as an analysis tool, having very little impact on the fully-tethered load cell measurements.
